# Use of functional magnetic resonance imaging to identify cortical loci for lower limb movements and their efficacy for individuals after stroke

**DOI:** 10.1186/s12984-024-01319-8

**Published:** 2024-04-16

**Authors:** Minseok Choi, Hyun-Chul Kim, Inchan Youn, Song Joo Lee, Jong-Hwan Lee

**Affiliations:** 1 Department of Brain and Cognitive Engineering, Korea University, Seoul, South Korea; 2 Department of Artificial Intelligence, Kyungpook National University, Daegu, South Korea; 3 Bionics Research Center, Biomedical Research Division, Korea Institute of Science and Technology, Seoul, South Korea; 4 Interdisciplinary Program in Precision Public Health, Korea University, Seoul, South Korea; 5 McGovern Institute for Brain Research, Massachusetts Institute of Technology, Boston, Massachusetts USA

**Keywords:** Functional MRI, Lower limb, Multivoxel pattern analysis, Paracentral lobule, Representational similarity analysis

## Abstract

**Background:**

Identification of cortical loci for lower limb movements for stroke rehabilitation is crucial for better rehabilitation outcomes via noninvasive brain stimulation by targeting the fine-grained cortical loci of the movements. However, identification of the cortical loci for lower limb movements using functional MRI (fMRI) is challenging due to head motion and difficulty in isolating different types of movement. Therefore, we developed a custom-made MR-compatible footplate and leg cushion to identify the cortical loci for lower limb movements and conducted multivariate analysis on the fMRI data. We evaluated the validity of the identified loci using both fMRI and behavioral data, obtained from healthy participants as well as individuals after stroke.

**Methods:**

We recruited 33 healthy participants who performed four different lower limb movements (ankle dorsiflexion, ankle rotation, knee extension, and toe flexion) using our custom-built equipment while fMRI data were acquired. A subgroup of these participants (Dataset 1; *n* = 21) was used to identify the cortical loci associated with each lower limb movement in the paracentral lobule (PCL) using multivoxel pattern analysis and representational similarity analysis. The identified cortical loci were then evaluated using the remaining healthy participants (Dataset 2; *n* = 11), for whom the laterality index (LI) was calculated for each lower limb movement using the cortical loci identified for the left and right lower limbs. In addition, we acquired a dataset from 15 individuals with chronic stroke for regression analysis using the LI and the Fugl–Meyer Assessment (FMA) scale.

**Results:**

The cortical loci associated with the lower limb movements were hierarchically organized in the medial wall of the PCL following the cortical homunculus. The LI was clearer using the identified cortical loci than using the PCL. The healthy participants (mean ± standard deviation: 0.12 ± 0.30; range: – 0.63 to 0.91) exhibited a higher contralateral LI than the individuals after stroke (0.07 ± 0.47; – 0.83 to 0.97). The corresponding LI scores for individuals after stroke showed a significant positive correlation with the FMA scale for paretic side movement in ankle dorsiflexion (R^2^ = 0.33, *p* = 0.025) and toe flexion (R^2^ = 0.37, *p* = 0.016).

**Conclusions:**

The cortical loci associated with lower limb movements in the PCL identified in healthy participants were validated using independent groups of healthy participants and individuals after stroke. Our findings suggest that these cortical loci may be beneficial for the neurorehabilitation of lower limb movement in individuals after stroke, such as in developing effective rehabilitation interventions guided by the LI scores obtained for neuronal activations calculated from the identified cortical loci across the paretic and non-paretic sides of the brain.

## Background

Functional magnetic resonance imaging (fMRI) has been widely used to investigate the motor functions of the human brain, particularly upper limb movements such as finger tapping and hand grasping/clenching. Distinct cortical loci have been identified for different types of upper limb movement [1–3], and these cortical loci have been successfully employed for the neurorehabilitation/neuroplasticity of individuals in the chronic stage after stroke [4–6]. The importance of identifying the cortical loci for lower limb movement in stroke rehabilitation has been discussed in previous studies [7, 8]. For example, noninvasive brain stimulations such as repetitive transcranial magnetic stimulation (rTMS) and transcranial direct current stimulation (tDCS) have been demonstrated to be effective in improving the gait and balance performance of individuals with subacute and chronic stroke by targeting the cortical loci, including the primary motor cortex (M1) [7]. We believe rehabilitation outcomes would be further enhanced by targeting the stimulation of fine-grained cortical loci for lower limb movement.

In this context, previous studies have investigated neuronal patterns observed from fMRI during lower limb movements [9–12]. For example, Luft and colleagues (2002) compared brain activations between upper and lower limb movements by incorporating finger, elbow, and knee movements in their lateralization index (LI) across various regions-of-interest (ROIs), including the M1, primary somatosensory cortex (S1), primary motor area (SMA), and cerebellum [10]. Kapreli et al. extended these findings by including the ankle and toes to differentiate the LI of brain activations for finger movement from that for the movement of lower limb joints [11, 12] and by combining movements across the ankle, knee, and hip [9]. These previous studies have generally reported different activation loci for lower limb movements compared with upper limb movements and overlapping spatial layouts for neuronal activations across lower limb movements. Another line of research has compared the neuronal activations of imagined lower limb movements with executed and/or observed movements for the right ankle [13], foot-kicking [14], and stepping [15].

However, few studies have investigated the distinct cortical loci considering hierarchical representations in the cortical homunculus for the movement of the ankle, toe, or knee, which are feasible movements of the lower limb extremities for fMRI acquisition because head motion is potentially more controllable compared with hip joint movement [11]. The identification of cortical loci specific to these lower limb movements in the median wall of the sensorimotor area mainly in the paracentral lobule (PCL) region is more challenging than for upper limb movements because the motor cortex associated with the lower limbs is smaller in volume than that for the upper limbs based on the cortical homunculus [16]. In addition, isolation of individual lower limb movements is more demanding due to the potentially greater head motion [10, 17–19].

Previous studies have investigated the neuronal activation patterns of lower limb movements based on the guidance of MR-compatible equipment. These studies include the identification of neuronal activation patterns for active and passive stepping movements [15, 20, 21] and pedaling [18] and the real-time monitoring of ankle, knee, and hip torques with their associated neuronal activations [22]. In the present study, we developed a custom-made MR-compatible footplate and leg cushion to isolate individual lower limb movements and minimize potential head motion during fMRI data acquisition. We then identified the cortical loci for lower limb movements using fMRI data acquired from healthy participants and subsequent multivariate analysis. Conventionally, it is not easy to delineate these loci due to the constrained cortical regions, particularly in the PCL, which is the medial continuation of the precentral and postcentral gyri [23], and overlapping functional territories across the lower limbs. We also investigated the efficacy of the cortical loci identified from healthy participants for individuals with chronic stroke. We hypothesized that our custom-built equipment and multivariate analytical methods would be useful for identifying hierarchically organized cortical loci for lower limb movements (i.e., an inferior location for the toe to a superior location for the knee). We also hypothesized that the identified cortical loci would be useful in evaluating the neural features of individuals with chronic stroke.

## Methods

### Overview

Using the fMRI data acquired from healthy participants by utilizing a custom-built MR-compatible footplate and leg cushion, we identified the cortical loci for lower limb movements using multivariate analyses of multivoxel pattern analysis (MVPA) [24] and representational similarity analysis (RSA) [25]. The laterality index (LI) was then calculated for each individual lower limb movement based on the identified cortical loci using the neuronal activations in the left and right hemispheres. Finally, we investigated the potential use of the LI to explain behavioral data for the lower limb movements of individuals with chronic stroke.

### Participants

The Institutional Review Board (IRB) of Korea University and the Korea Institute of Science and Technology (KIST) approved this study. Participants provided written informed consent and were compensated in accordance with the IRB document. Thirty-three healthy participants and 15 individuals with chronic stroke volunteered to participate. The data from 21 of the 34 healthy participants (healthy dataset 1) were used to identify the cortical loci for lower limb movements. The data from the 11 healthy participants (healthy dataset 2) and 15 individuals with chronic stroke whose lower limb movement was compromised by stroke (stroke dataset) were used to evaluate the validity of the identified functional loci. Additionally, 11 healthy participants (equipment evaluation dataset) were recruited to evaluate the validity of our MR-compatible footplate and knee cushion to reduce head motion. Participants were recruited from both Korea University and KIST and all the MRI data were acquired at Korea University Brain Imaging Center. The number of participants was aimed to have at least 12 participants for the healthy group and 15 participants for the stroke group, which is in line with the recent report on a median sample size of 12 for highly cited experimental fMRI studies with a single group and 14.5 for highly cited clinical fMRI studies with a patient group [26]. We also conducted a power analysis based on a *t*-test with a Type I error of 0.8, a power level (1—Type II error) of 0.8, and an alpha of 0.05 which was obtained as 11 using the G*power toolbox [27, 28]. The data collection periods were Sep 09, 2020–Apr 28, 2021, for healthy dataset 1; Sep 24, 2020–Jun 03, 2021, for healthy dataset 2; Jun 01, 2021–Sep 13, 2022, for the stroke dataset; and May 13, 2021–May 27, 2021, for the equipment evaluation dataset.

We pre-screened volunteers via a telephone interview to ensure that they met the inclusion criteria of no history of neuropsychiatric medication or physical/mental disorders. Additionally, volunteers with claustrophobia and those who had undergone a surgical procedure for implant devices were excluded. We did not consider a specific age range or gender distribution in the inclusion criteria to facilitate participant recruitment. Those who passed this pre-screening underwent a face-to-face interview, during which we assessed their mental state and cognitive abilities using the Korean version of the Mini-Mental State Examination (MMSE-K), the Beck Depression Inventory (BDI), the Beck Anxiety Inventory (BAI), the Big Five Inventory-10 (BFI-10), and the Patient Health Questionnaire (PHQ) [29–33]. Handedness and footedness scores were obtained on the day of the MRI session using the Edinburgh Handedness Inventory and the Waterloo Footedness Questionnaire-Revised (WFQ-R) [34, 35]. Detailed information for each group is presented in Table 1. We also utilized the Fugl–Meyer Assessment (FMA) scores [36], a widely accepted evaluation tool for assessing upper and lower limb motor function during stroke rehabilitation. Occupational therapists widely recognize and use these scores for their intertester/interrater reliability and validity [37–39] and we therefore employed them in our study. A licensed physical therapist administered the assessment to individuals with chronic stroke. These individuals were recruited solely from KIST and their corresponding FMA scores were obtained.

**Table 1:** Demographic, behavioral, and psychological information for the participants

	Healthy 1 (*n* = 21, 16 males)	Healthy 2 (*n* = 11, 6 males)	Stroke (*n* = 15, 12 males)	Equipment (*n* = 11, 5 males)
Age	26.0 ± 2.9	54.1 ± 24.4	58.1 ± 10.6	22.7 ± 3.5
WFQ-R	6.2 ± 8.7	11.0 ± 7.4	-1.3 ± 15.9	5.7 ± 10.7
EHI	57.3 ± 50.0	90.0 ± 14.6	8.2 ± 93.8	39.0 ± 61.3
BFI-10				
Extraversion	5.0 ± 1.3	6.9 ± 1.6	5.6 ± 1.5	5.9 ± 1.9
Agreeableness	6.7 ± 1.7	7.5 ± 2.1	8.0 ± 1.7	7.4 ± 1.1
Conscientiousness	5.6 ± 1.5	7.5 ± 2.0	8.0 ± 2.6	6.0 ± 1.8
Neuroticism	5.6 ± 1.4	5.0 ± 2.1	4.7 ± 2.6	6.1 ± 2.2
Openness to experience	6.8 ± 1.4	6.9 ± 1.4	7.3 ± 2.1	7.6 ± 1.2
PSS	15.5 ± 5.2	10.9 ± 4.6	12.7 ± 6.2	12.9 ± 7.4
BDI	6.1 ± 5.9	2.0 ± 1.5	10.0 ± 11.8	6.2 ± 7.6
BAI	4.4 ± 5.9	1.3 ± 1.5	5.5 ± 6.5	3.6 ± 4.2

Healthy 1, Healthy dataset 1; Healthy 2, Healthy dataset 2; Stroke, Stroke dataset; Equipment, Equipment evaluation dataset; WFQ-R, Waterloo Footedness Questionnaire-Revised; BFI-10, Big Five Inventory-10; EHI, Edinburg Handedness Inventory; PSS, Perceived Stress Scale; BDI, Beck Depression Inventory; BAI, Beck Anxiety Inventory

### Experiment paradigm

**Fig. 1:**
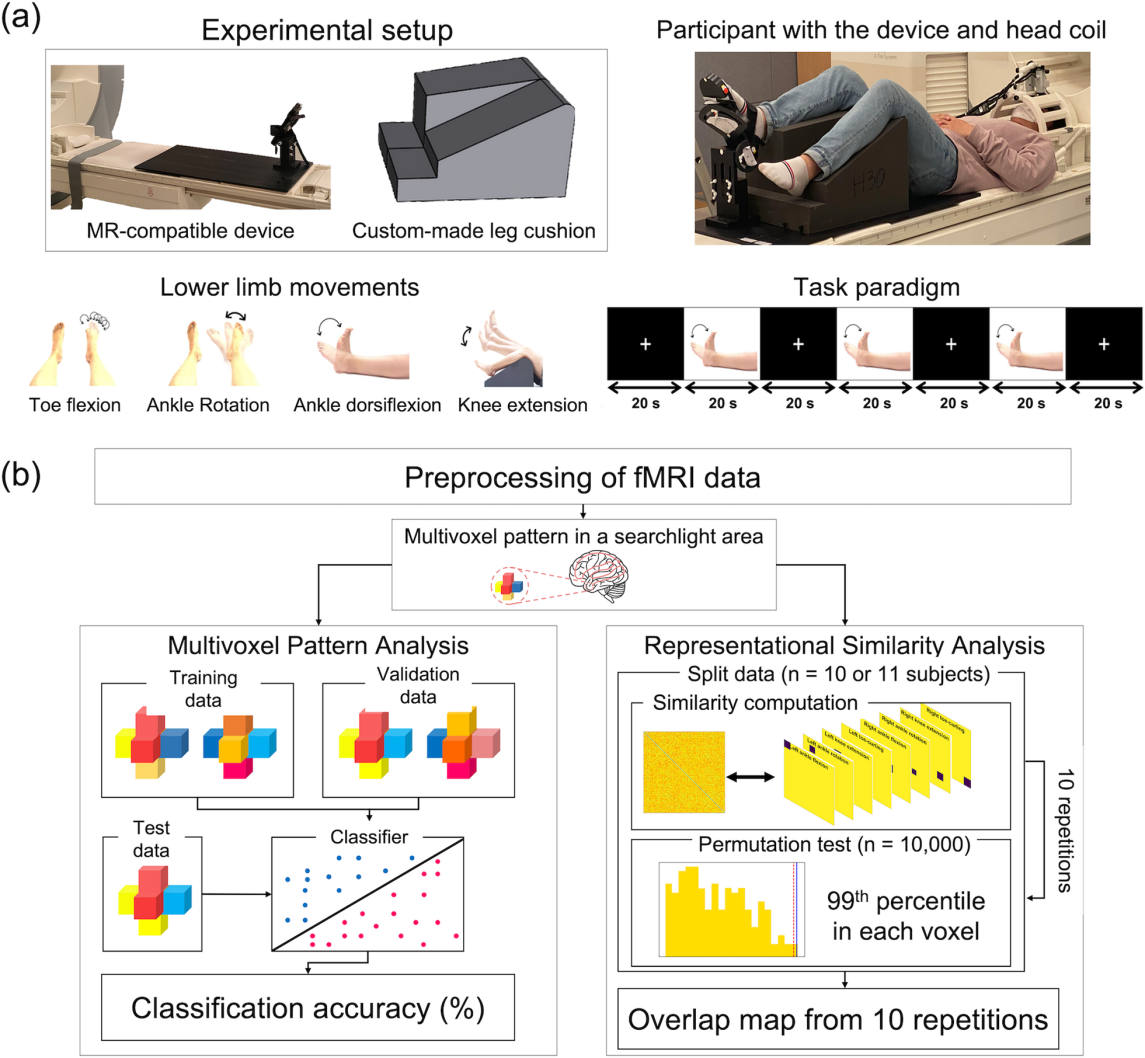
Overview of the experiment and analysis setup. **a** Our custom-made MR-compatible footplate and leg cushion (top) and experiment setup for the lower limb movement tasks. Illustrations guiding each of the four movements and an example task paradigm for the ankle dorsiflexion task (bottom). **b** Analysis framework for multivoxel pattern analysis (left) and representational similarity analysis (right)

Figure 1 illustrates the experiment setup and task paradigm. Participants lay on the scanner bore in a supine position, and their head was secured with a custom memory foam cushion to reduce head motion during lower limb movements. Participants were instructed to perform four lower limb movements on each side (knee extension, ankle dorsiflexion, ankle rotation, and toe flexion) during fMRI acquisition using our MR-compatible custom-made footplate, which was developed to isolate and guide the movements. All of the participants were trained on how to use the footplate and performed each lower limb movement five times before the MRI session. During the MRI session, participants performed each lower limb movement in one fMRI run, with eight fMRI runs acquired across the four lower limb movements for the left and right legs. Each fMRI run consisted of three task blocks (20 s/block) interleaved by resting/fixation blocks (20 s/block). The order of the eight lower limb movements was counterbalanced for each subject by utilizing Python’s random shuffling function (i.e., 'numpy.random.shuffle') first to select either the right or left leg and then select the movement order for the four lower limbs. All of the individuals after stroke completed two out of the four lower limb movements (ankle dorsiflexion and toe flexion) and six individuals after stroke completed all four lower limb movements on both sides.

### fMRI data acquisition and preprocessing

fMRI data were acquired using a 3-T Tim-Trio scanner with a 12-channel head coil (Siemens, Erlangen, Germany). A standard gradient-echo echo-planar imaging (EPI) pulse sequence (repetition time/echo time = 1,440/30 ms; flip angle = 71°; field-of-view = 192 mm × 192 mm; 50 axial slices without a gap; voxel size = 3 mm × 3 mm × 3 mm; multiband factor of two and GeneRalized Autocalibrating Partially Parallel Acquisition [GRAPPA]) was used to measure the blood-oxygenation-level-dependent (BOLD) contrast arising from the neural activations. The EPI parameters were modified during fMRI acquisition for the individuals after stroke to enhance the signal-to-noise ratio of the BOLD contrast (repetition time/echo time = 2000/30 ms; flip angle = 84°; field-of-view = 240 mm × 240 mm; 35 axial slices without a gap; voxel size = 3 mm × 3 mm × 3 mm; multiband factor of one and GRAPPA). The T1-weighted structural MRI volume was acquired using a magnetization-prepared rapid gradient-echo (MPRAGE) pulse sequence (repetition time/echo time = 1900/2.28 ms; flip angle = 8°; field-of-view = 256 mm × 256 mm; voxel size = 1 mm × 1 mm × 1 mm).

The fMRI data were preprocessed using AFNI software (afni.niml.nih.gov) in the following order: slice-timing correction, realignment of fMRI volumes in each run to the first volume, and spatial normalization to the Montreal Neurological Institute (MNI) space using a non-linear registration function with 3 mm × 3 mm × 3 mm voxel-size resampling. In the slice-timing correction process, the acquisition timing for all of the slices within a volume was aligned to the acquisition time for the first slice using Fourier order/function-based temporal interpolation. In the realignment of the fMRI volumes, the first EPI volume was used as a target volume, and the remaining volumes in a run were aligned to the target volume to correct for potential head motion during the run. For the three blocks in each fMRI run, a task block was excluded if greater than 10% of the total number of volumes in the block exhibited severe head motion (i.e., a Euclidean distance greater than 0.5 mm) in accordance with the guidelines provided by previous work [40]. When the three blocks for each of all eight tasks/runs passed this head motion criterion, the dataset of the corresponding subject was included in the analysis. Subsequently, 13 out of the totally recruited 34 subjects in the Healthy 1 group were excluded. When we noticed the relatively high dropout rate from the Healthy 1 group, we informed the participants in the Healthy 2 and Stroke groups about the importance of reducing head motion during fMRI data acquisition. We also monitored their head motion after each fMRI run was acquired and asked the participants to perform the run again if the head motion threshold was exceeded. One individual after a stroke could not finish the experiment due to claustrophobia. Spatial normalization was conducted using 21 parameter linear affine transformation (with the “3dAllineate” command) followed by nonlinear warping (the “3dQwarp” command). The linear trend due to scanner instability, including eddy current distortions, was removed using polynomial function detrending (with the “3dDeconvolve” command).

### Evaluation of MR-compatible devices

We built a custom-made footplate and leg cushion to reduce potential head motion during lower limb movement tasks and to isolate and guide each of the ankle and toe movements. The success of the devices in reducing head motion was evaluated using fMRI data in the equipment evaluation dataset. Specifically, we asked the corresponding subjects to perform ankle dorsiflexion and knee extension twice with and without our custom-made footplate and leg cushion because we observed that the head motion was largest for these two movements. We then applied Welch’s *t*-tests to the six head motion parameters estimated from the realignment step in the preprocessing of the fMRI data. The corresponding statistical significance was investigated based on multiple comparison correction using Tukey–Kramer tests for unequal sample sizes.

### General linear model to estimate neuronal activations

General linear model (GLM) analysis was carried out using the fMRI data from each subject to estimate the neuronal activations induced by the individual lower limb movements. Specifically, a reference BOLD signal was obtained by convolving a canonical hemodynamic response function with a boxcar model for each task block and used as a task-related regressor for the block [41]. Thus, each fMRI run had three task-related regressors. Six head motion parameters were included as nuisance regressors to remove confounding artifacts in the BOLD signal due to head movement [42, 43]. A restricted maximum likelihood-based estimation was used to obtain a beta-valued map associated with each task block as neuronal activations [44]. The resulting beta-valued maps from the subjects in the Healthy 1 group were subject to repeated measures analysis-of-variance (ANOVA) across the four tasks for each side of the lower limbs within the univariate analysis framework. As a result, the main effect of the four movements was obtained (uncorrected *p*-value < 0.05).

### Multivoxel pattern analysis (MVPA) of four lower limb movements

**Fig. 2:**
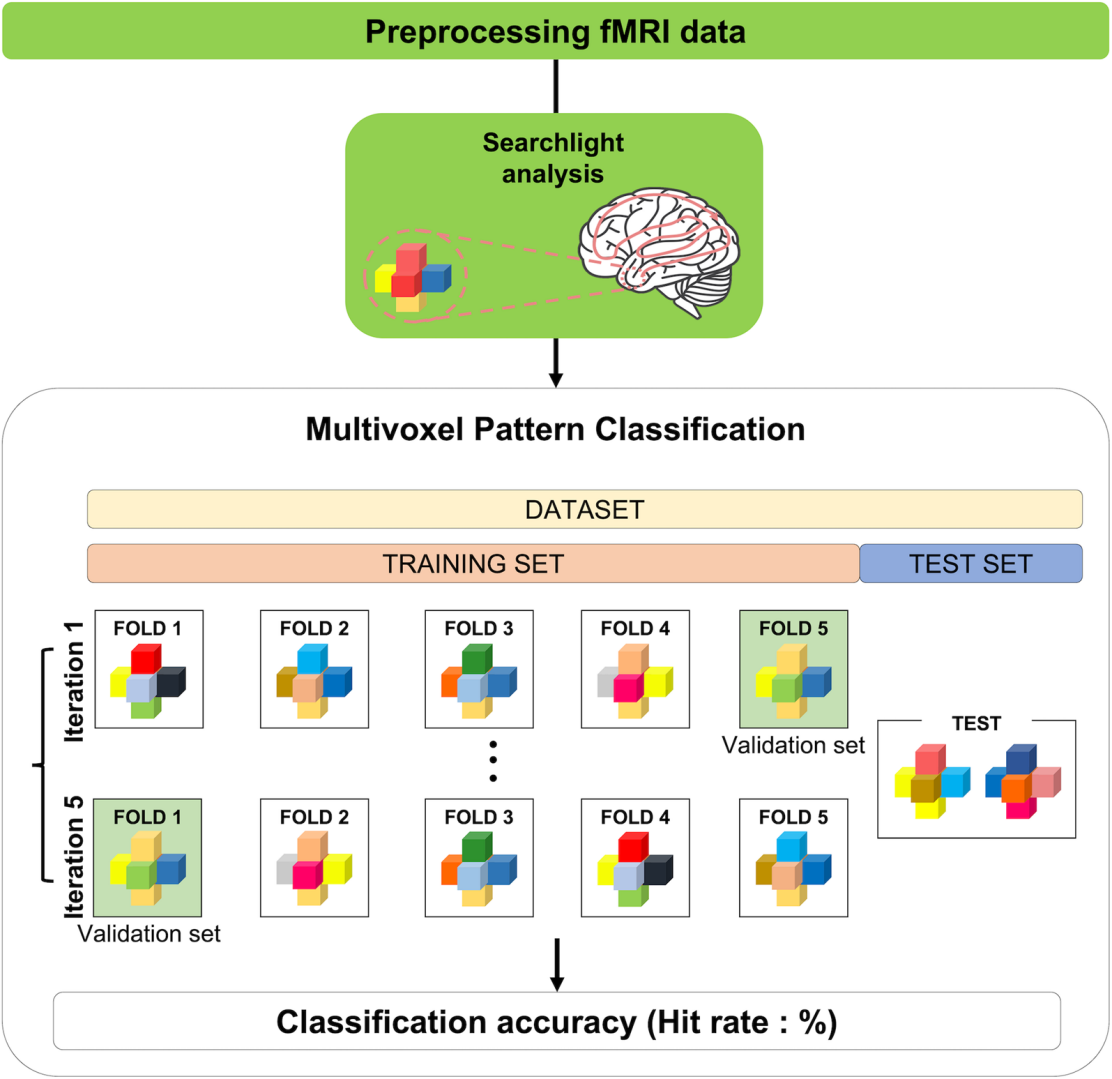
Overview of multi-voxel pattern analysis with five-fold cross-validation

We identified brain regions in the PCL with distinct activation patterns for each of the four lower limb movements in each leg using the healthy dataset 1. To this end, the PCL area was defined using an automated anatomical labeling atlas [45], and multivoxel patterns with beta values in the PCL were classified into one of the four lower limb movements. We used a support vector machine (SVM) with either a linear or non-linear (i.e., radial basis function [RBF]) kernel as a classifier to determine the task information. A nested five-fold cross-validation scheme was adopted in which the beta maps from the 21 subjects were stratified into training, validation, and test data using the subjects' indices (Fig. 2). The validation data were used to optimize the hyperparameters for the SVM models, including the regularization parameter *C* for linear/non-linear SVMs and variance γ of the RBF kernel for the non-linear SVM. The candidate values for both *C* and γ had a range of 10^–4^ to 10^3^ with power of 10 intervals (i.e., 10^–4^, 10^–3^, …, 10^2^, 10^3^). The optimal hyperparameters were then selected from a grid search by considering all of the combinatorial values of *C* and γ for the non-linear SVM. The classification performance was evaluated across three MVPA scenarios to determine the optimal performance and their conditions: (1) three beta-valued maps corresponding to the three blocks in each run vs. one average beta-valued map across the three blocks in each run to evaluate the block- or run-wise performance; (2) without or with spatial smoothing using a 4-mm full-width at half-maximum Gaussian kernel to evaluate the performance with additional spatial smoothing preprocessing; and (3) a sphere radius of 1, 2, or 3 voxels to define the multivoxel patterns for the searchlight to evaluate the performance depending on the multivoxel pattern dimensions.

### Representational similarity analysis (RSA) to identify functional loci

**Fig. 3:**
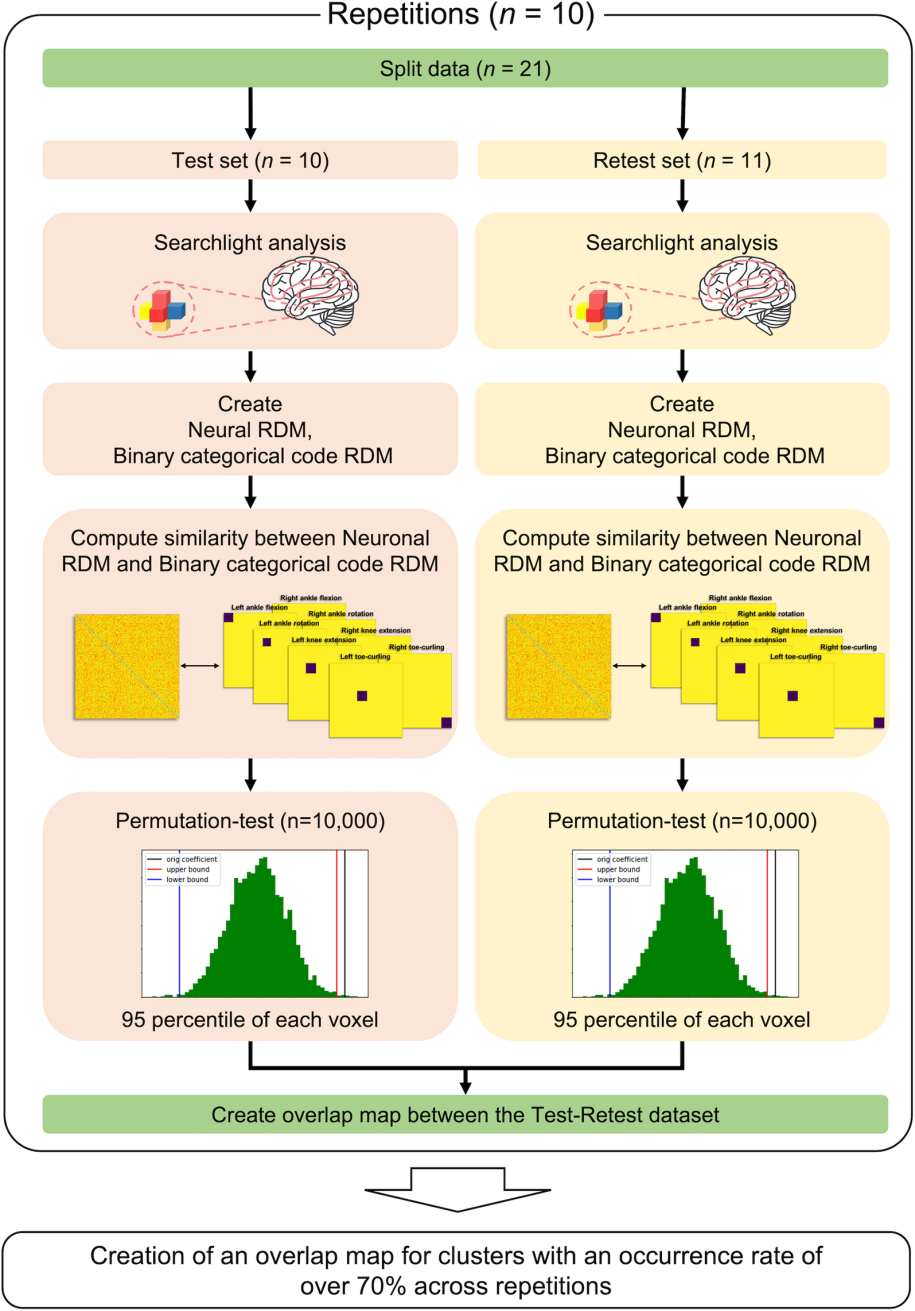
Overview of representational similarity analysis with test–retest-based cross-validation

We conducted an RSA to pinpoint the cortical loci of each lower limb movement. To this end, we calculated (a) a neural representational dissimilarity matrix (RDM) using neuronal activation patterns across the eight tasks and (b) a non-neural binary categorical RDM using code patterns to define each movement of interest among the four lower limb movements (Fig. 3). RSA was then conducted to identify the neural representation of each lower limb movement based on the similarity of holistic geometric representations reflected in the two RDMs. In detail, the neural RDM for each voxel in the PCL was defined from the neighboring multivoxel patterns in the sphere with a three-voxel radius (i.e., 123 voxels). The dissimilarity value was defined as “1 – the Pearson’s correlation coefficient” of the pairwise multivoxel patterns with beta-valued maps from the GLM in the sphere across the fMRI runs (i.e., tasks) without any thresholding/normalization. Thus, the resulting dissimilarity value had a minimum of 0 (exactly the same pattern) and a maximum of 2 (exactly the opposite pattern). The non-neural code RDM (with 1 being the most dissimilar [i.e., different tasks] and 0 being the most similar [i.e., the same task]) was also constructed to encode the information across the eight tasks. The similarity between the neural RDM and non-neural code RDM was calculated using Spearman's rank correlation (ρ).

We evaluated the test–retest reliability of the RSA results. To this end, we divided the 21 subjects randomly into 10 and 11 subjects, and two sets of RSA were conducted on the data from these test and retest subjects. To define the significant voxel clusters in each of two RSA results, the statistical significance of the similarity value was corrected based on a null distribution of the similarity values drawn from the 10,000 randomly shuffled movement indices across the fMRI runs/tasks (i.e., corrected *p* < 0.01 from 10,000 random permutations) [46]. We identified the significant voxel clusters that represented the overlap between the two RSA results. This test–retest-based RSA was conducted 10 times using the 10 sets of randomly selected sub-groups with 10 or 11 subjects. Finally, the overlapping significant voxel clusters that occurred more than seven times out of the 10 repetitions were identified (minimum of five-voxel clusters).

### Evaluation of cortical loci using the laterality index (LI)

We employed the AveLI method, a robust unbiased threshold-free LI for the fMRI data [47], to evaluate the validity of the identified cortical loci compared with the anatomically defined PCL area. The AveLI is computed as the average of all of the LIs using the adaptive threshold to define the voxels with neuronal activations [47]:

$$AveLI=\frac{\sum ({subLI}_{i})}{VN},$$

where *VN* is the total number of voxels with positive *t*-scores. ${subLI}_{i}$ is the LI defined using the *i*th voxel with a positive *t*-score as the threshold:

$${subLI}_{i}=\frac{{Rt}_{i}-{Lt}_{i}}{{Rt}_{i}+{Lt}_{i}},$$

where ${Rt}_{i}$ and ${Lt}_{i}$ are the sum of the *t*-scores in the contralateral hemisphere for right-side movement and for left-side movement, respectively. The AveLI values were obtained for healthy dataset 1, healthy dataset 2, and the stroke dataset. The lower limbs of the individuals after stroke were affected either on their left or right side (i.e., the paretic side). Thus, the calculation of ${subLI}_{i}$ was modified as follows:

$${subLI}_{i}^{Stroke}=\frac{{NPt}_{i}-{Pt}_{i}}{{NPt}_{i}+{Pt}_{i}},$$

where ${NPt}_{i}$ and ${Pt}_{i}$ are the sum of *t*-scores in the contralateral hemisphere for non-paretic side movement and for paretic side movement, respectively.

Consequently, we conducted linear regression analysis to investigate the association between the AveLI and behavioral FMA data for the individuals after stroke. We applied a permutation test (*n* = 10,000) to correct the significance of the regression analysis (*p* < 0.05) based on the estimated null distribution and bootstrap resampling (*n* = 10,000) to estimate the confidence interval for the regression line [43].

## Results

### Evaluation outcomes for MR-compatible devices

**Fig. 4:**
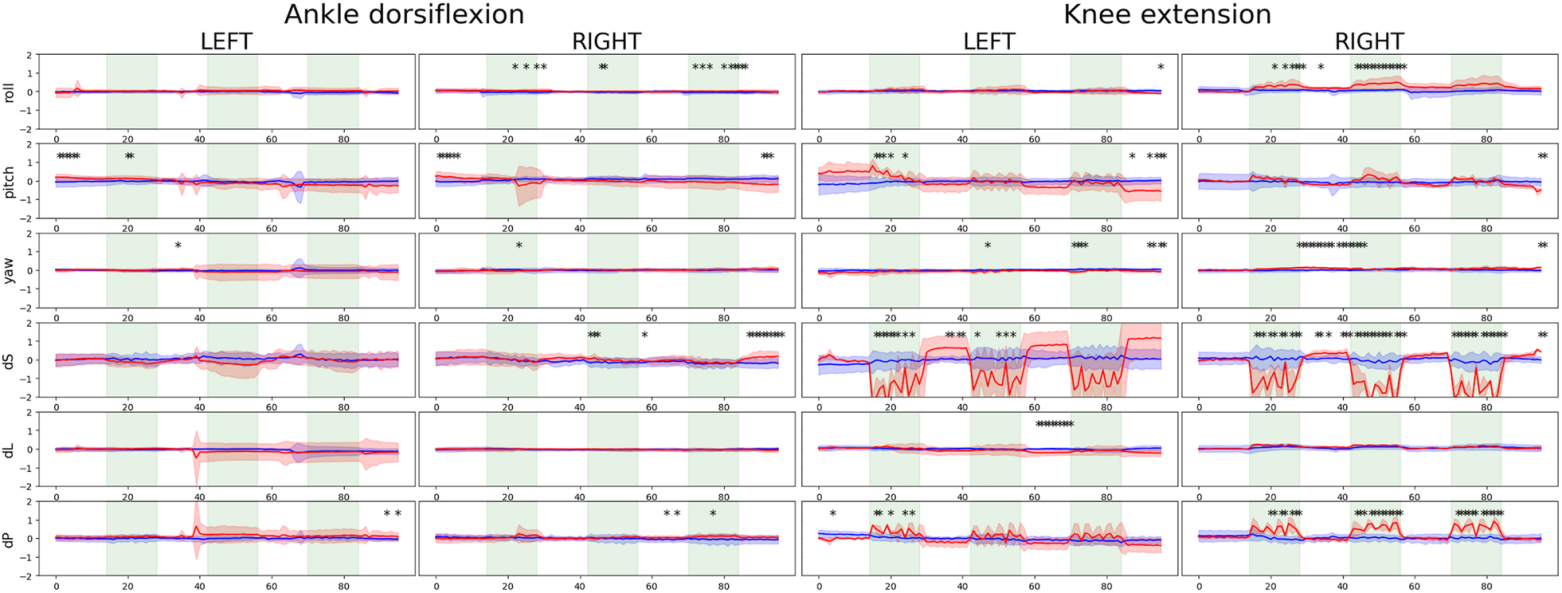
Mean and standard deviation of head motion by participants estimated using the motion correction step in fMRI preprocessing. The red line and blue shading indicate cases where the custom-made MR-compatible footplate device and custom-made leg cushion were not used during the ankle dorsiflexion and knee extension movements. The time point when the head motion without the footplate device and leg cushion was higher compared to the head motion with the footplate device and leg cushion is marked (^*^p < 0.05 corrected using Tukey–Kramer tests)

Figure 4 presents the mean and standard deviation for the head motion parameters estimated from the measured fMRI data with and without equipment using the equipment evaluation dataset (*n* = 11; evaluated using paired *t*-tests with a *p*-value < 0.05 corrected using Tukey–Kramer tests for unequal sample sizes). Overall, when participants performed lower limb movements with the MR-compatible custom-made footplate and leg cushion, head motion was substantially reduced during the knee extension movement, particularly in terms of roll, displacement in the posterior (dP) direction, and displacement in the superior (dS) direction.

### Univariate analysis results for neuronal activations across the four lower limb movements

**Fig. 5:**
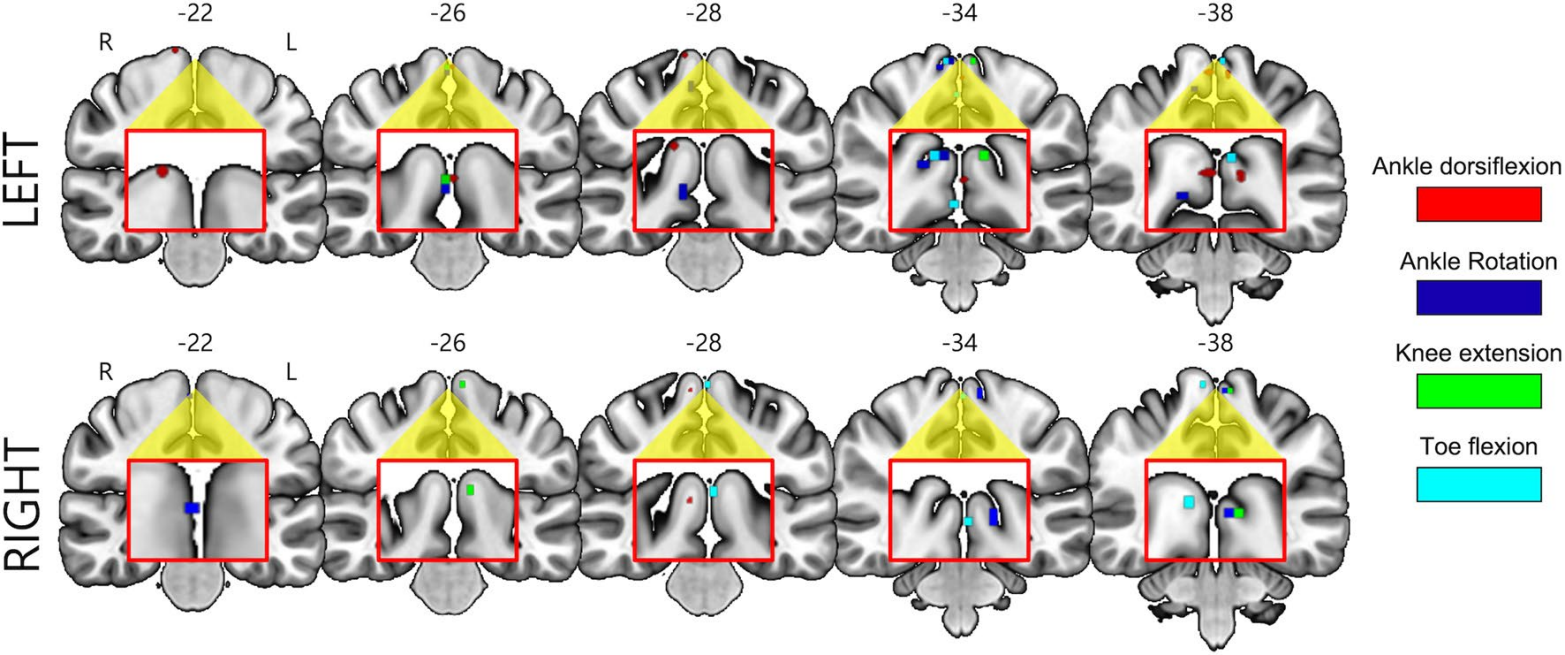
Statistical maps from repeated measures ANOVA across the four lower limb movements of each leg using the corresponding beta-valued maps from the general linear model with healthy dataset 1 (uncorrected *p*-value < 0.05)

Figure 5 shows the neuronal activations obtained from the one-way ANOVA for each side of the leg using healthy dataset 1 (uncorrected *p*-value < 0.05). Overall, the identified clusters across the lower limb movements did not follow the cortical homunculus. Furthermore, all clusters disappeared when we corrected the *p*-value using least stringent random-permutation-based multiple comparison correction.

### MVPA classification of the four lower limb movements

**Fig. 6:**
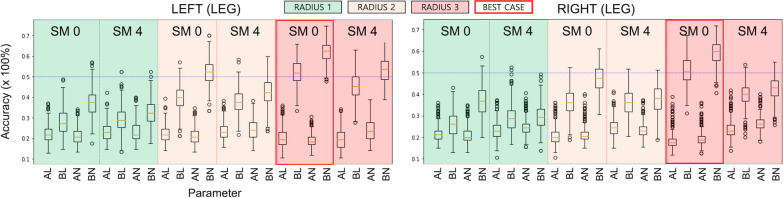
MVPA classification results. The accuracy is summarized as box-whisker plots based on the SVM kernel type (linear or nonlinear), searchlight size (1-, 2-, or 3-voxel radius), and spatial smoothing kernel size (i.e., 0 or 4 mm). On the x-axis, “A” denotes the averaged beta map for three blocks as input, “B” denotes block-wise beta maps as input, “L” represents the linear kernel SVM, and “N” is the non-linear RBF kernel SVM. SM, smoothing kernel size; RADIUS, radius of the searchlight sphere

**Fig. 7:**
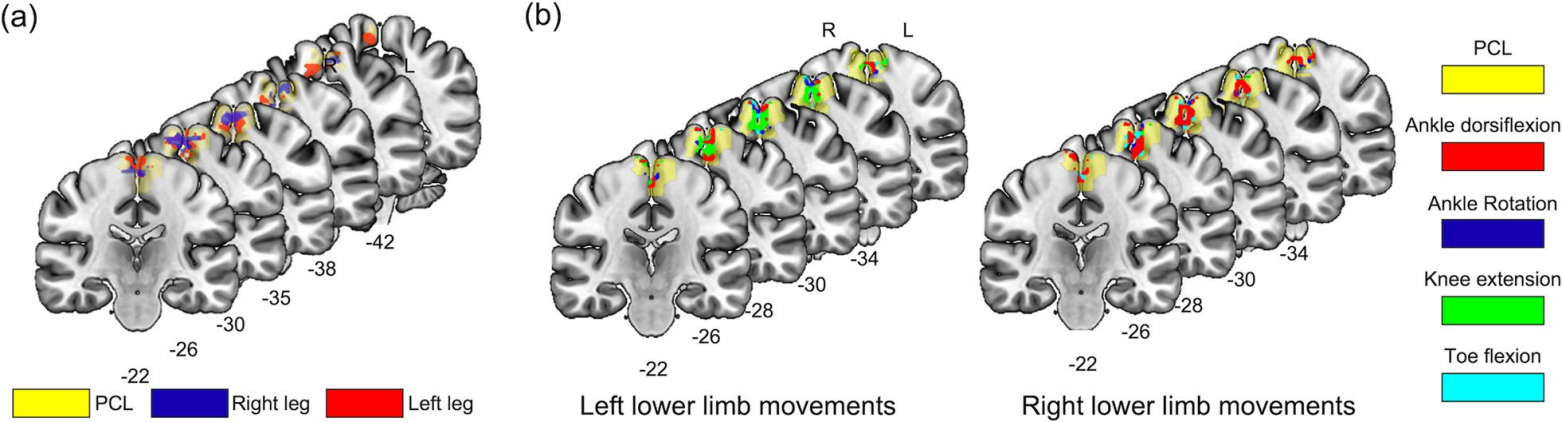
Subregions in the PCL for lower limb movement identified from the MVPA and RSA. **a** The results from the MVPA classification in the paracentral lobule (yellow). The red and blue regions are voxel clusters that had a classification accuracy of at least 65% (red denotes the movement of the left lower limb, while blue represents the right). **b** The results from the RSA in the PCL (yellow). The cortical loci for each lower limb movement is color-coded (cyan for toe flexion, red for ankle dorsiflexion, blue for ankle rotation, and green for knee extension)

Figure 6 presents the accuracy of the MVPA classification for the four lower limb movements across various parameters using healthy dataset 1 (*n* = 21). Overall, when (i) block-based input beta-maps were used without spatial smoothing; and (ii) a searchlight radius size of 3 for the non-linear RBF-kernel SVM was used for the MVPA (red box), the corresponding performance was superior to the SVM models with alternative parameters (e.g., average-based input beta-valued map with a searchlight radius size of 1 or 2, linear kernel SVM). Figure 7a illustrates the voxel clusters whose classification accuracy was well above (> 65%) the level of chance (25%).

### Cortical loci of the four movements from RSA

Figure 7b illustrates the cortical loci across the four lower limb movements, with the corresponding MNI coordinates presented in Table 2. Specifically, the locus for toe flexion (cyan) was located at the most inferior location compared to that of knee extension (green). The cortical loci for the ankle movements (red and blue) were located between the toe and knee movements, particularly for the left lower limb. The cortical loci were primarily located in the hemispheres contralateral to the movement.

**Table 2:** Functional loci identified for the four lower limb movements on each side based on RSA (the x, y, and z coordinates are in the MNI space)

Task	Coordinate			Size
	x	y	z	
Left lower limb				
Ankle dorsiflexion	+ 4.5	+ 25.5	+ 67.5	11
	+ 4.5	+ 25.5	+ 55.5	9
	+ 4.5	+ 34.5	+ 61.5	8
	–7.5	+ 28.4	+ 76.5	5
Ankle rotation	+ 1.5	+ 28.5	+ 55.5	61
	–4.5	+ 22.5	+ 73.5	5
Knee extension	+ 1.5	+ 25.5	+ 55.5	61
	–4.5	+ 22.5	+ 73.5	8
Toe flexion	+ 1.5	+ 28.5	+ 55.5	78
Right lower limb				
Ankle dorsiflexion	–1.5	+ 25.5	+ 52.5	52
	–4.5	+ 22.5	+ 73.5	8
Ankle rotation	–1.5	+ 28.5	+ 55.5	55
	–4.5	+ 22.5	+ 73.5	8
	+ 4.5	+ 28.5	+ 73.5	5
Knee extension	–1.5	+ 28.5	+ 55.5	62
	–4.5	+ 22.5	+ 73.5	8
Toe flexion	–1.5	+ 28.5	+ 55.5	60
	–4.5	+ 22.5	+ 73.5	8

RSA, representational similarity analysis; MNI, Montreal Neurological Institute

### Efficacy of identified cortical loci and laterality index (LI)

**Fig. 8:**
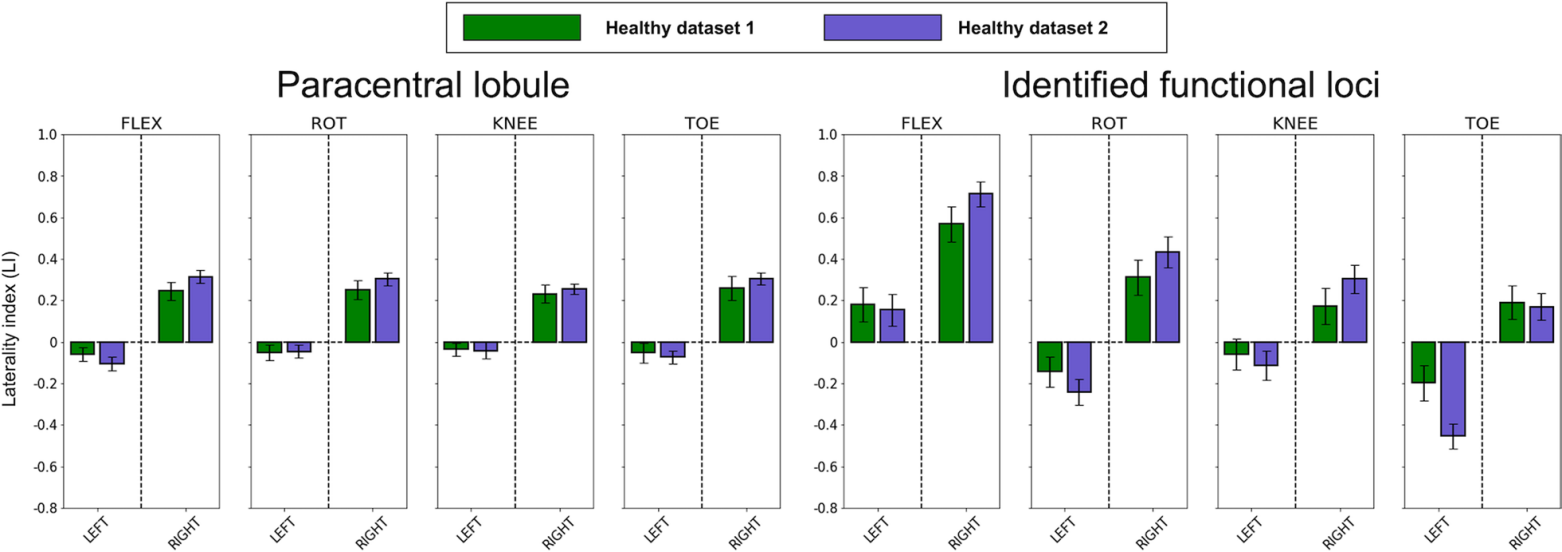
Laterality index comparison between the paracentral lobule and identified cortical loci across the four lower limb movements for the two healthy control groups

**Fig. 9:**
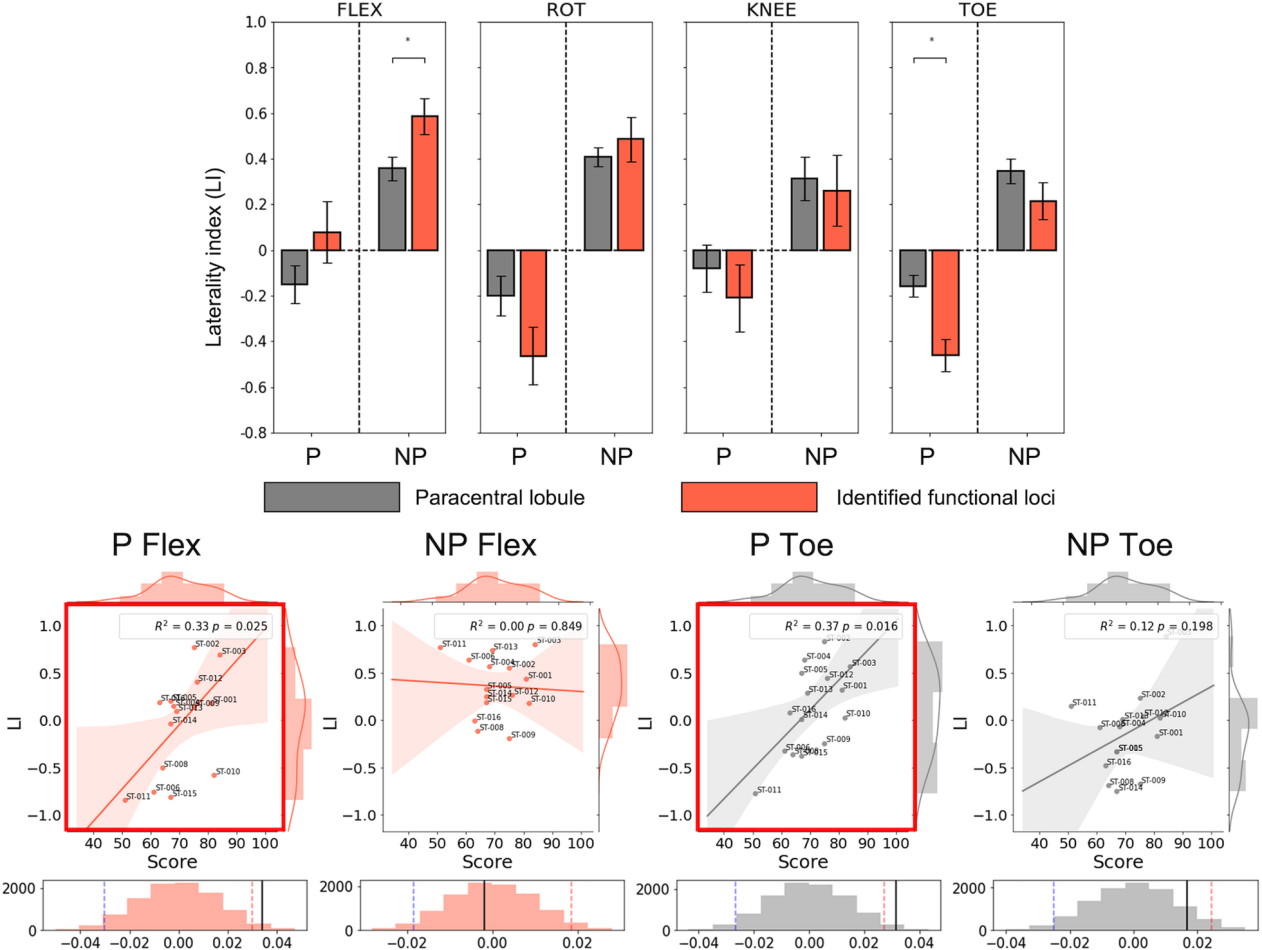
Laterality index comparison between the paracentral lobule and identified cortical loci across the four lower limb movements in individuals after stroke (^*^, *p*-value < 0.05 from paired *t*-tests). The scatter plots present the linear regression results for the AveLI scores and the FMA scale, with the significant results indicated by a red box (*p* < 0.05)

Figure 8 illustrates the AveLI values obtained from the healthy participants using the PCL area and using the identified cortical loci for the four lower limb movements. For both groups (i.e., healthy dataset 1 with *n* = 21 and dataset 2 with *n* = 11), the laterality indices were clearer when the identified cortical loci were used to calculate the LI in comparison to when the PCL area was used. A similar trend was also observed for the individuals after stroke (*n* = 15), in which the identified cortical loci had clearer LIs than the PCL (Fig. 9, top; ^*^ denotes *p*-value < 0.05 from a paired *t*-test). In addition, for the ankle dorsiflexion and toe flexion movements, there was a significant positive association between the LI and the FMA scale only on the paretic side (Fig. 9, bottom; *p*-value < 0.05 from 10,000 random permutations and the shaded region from 10,000 bootstrap resampling).

## Discussion

### Summary of the study

The main findings of the present study were as follows: (i) the cortical loci for ankle dorsiflexion, ankle rotation, knee movement, and toe flexion were hierarchically organized in the medial wall of the PCL based on data obtained from our custom-built MR-compatible equipment and multivariate analysis; (ii) healthy individuals exhibited a higher contralateral LI score for the lower limb movements than individuals after stroke; and (iii) the LI scores of individuals after stroke had a significant association with the FMA scale for ankle dorsiflexion and toe flexion. Our custom-made footplate and leg cushion successfully reduced head motion during the lower limb movements while fMRI data were acquired. The measured fMRI data were further analyzed using multivariate analysis techniques (i.e., MVPA and RSA). Consequently, the cortical loci for lower limb movement were identified in the medial wall of the PCL hierarchically distributed according to the cortical homunculus [48]. The topographic organization of the motor/somatotopy in the sensorimotor area as described by the cortical homunculus represents an important justification for our identified cortical foci. More specifically, the cortical loci for knee extension were located at the most superior-posterior position, while that for toe flexion was located at the most inferior position. The cortical loci for ankle movement were positioned between the cortical loci for knee extension and toe flexion. The classification accuracy for the four lower limb movements on each side, which was based on rigorous nested five-fold cross-validation with stratification based on healthy participants’ indices, was significantly higher (62.1 ± 4.6% for the left leg; 59.4 ± 5.7% for the right leg) than the level of chance (25%). The identified cortical loci were also gainfully applied to individuals after stroke, with the LI scores calculated using the cortical loci for left- and right-sided ankle dorsiflexion and toe flexion significantly correlated with the FMA scale from the paretic side of the individuals after stroke (i.e., R^2^ = 0.33, *p* = 0.026 for the paretic side in ankle dorsiflexion; R^2^ = 0.39, *p* = 0.013 for the paretic side in toe flexion).

### Cortical loci obtained from multivariate analysis

Both the MVPA and RSA are multivariate analysis techniques that consider multivoxel patterns for each center voxel, which is more advantageous than a univariate approach that considers only a single voxel due to the higher dimensionality of neuronal activations. Despite their similarity, the MVPA is more advantageous for the classification/prediction of multivoxel patterns [49], whereas the RSA is inherently more advantageous for the functional mapping of associated tasks/conditions based on multivoxel patterns [25]. Therefore, classification tests using MVPA have been instrumental in identifying brain regions associated with distinct task information in fMRI data [24, 50, 51], including the identification of the functional organization of body part movement in the motor and sensory cortices [52]. Our MVPA revealed the important sub-regions in the PCL that are highly representative of the four lower limb movements on each side. Despite the ability to identify the ROIs for the four lower limb movements in the PCL, the MVPA approach cannot easily pinpoint ROIs that are specific to individual lower limb movements. We thus deployed RSA, which enabled us to identify the lower limb-specific ROIs in the sub-regions of the PCL. However, no significant nor hierarchical cortical loci were found when we employed univariate analysis to distinguish neuronal activations across the four lower limb movements. The validity of the identified cortical loci for each lower limb was evaluated by comparing the corresponding LI scores between the cortical loci and the anatomical ROI (i.e., PCL). RSA has previously been successfully used to identify ROIs that are strongly associated with cognition and motor-related functions in the human brain using fMRI data [53, 54].

### Efficacy of identified cortical loci for lower limb movements for individuals after stroke

The cortical loci identified for the four lower limb movements in the present study represented the LI scores (i.e., the AveLI method described in “Evaluation of cortical loci using the laterality index (LI)” section) of the paretic and non-paretic lower limb movements better than did the PCL. Previous studies have reported overall elevated neuronal activations for paretic-side hand movements compared to non-paretic-side hand movements among individuals after stroke when compared with healthy controls [55, 56]. In addition, neuronal activations tend to exhibit ipsilaterally in the primary motor areas with paretic lower limb movements [57]. We observed that paretic-side lower limb movements produced a broader range of LI scores (from positive to negative) compared with non-paretic-side movements. In particular, for paretic-side movements, there was a strong positive association between the LI scores and FMA scale (i.e., an assessment tool for the upper and lower limb motor function of the affected side compared to the unaffected side during stroke rehabilitation) among individuals after stroke, meaning that those individuals after stroke with a higher score on the FMA scale (i.e., with greater physical ability) tended to exhibit ipsilateral neuronal activations, which has also been reported in previous studies [57]. On the other hand, individuals after stroke whose ability to move their lower limbs on the paretic side was weaker tended to exhibit contralateral neuronal activations. Our findings suggest that lower limb movement performance is strongly associated with neuronal activations. However, the reason why ipsilateral neuronal activations for lower limb movement on the paretic side tend to enhance physical performance needs to be addressed in future studies. One potential hypothesis is that neural plasticity occurs during the acute or sub-acute phase of stroke onset [58, 59], allowing the intact hemisphere of the motor area (i.e., the ipsilateral hemisphere for paretic-side movement) to quickly adopt the lower limb movement function for the paretic side because the brain tissue in the hemisphere contralateral to the paretic side has been compromised [60], which has also been reported for the upper limb movements for the individuals after stroke [61]. On the other hand, there was no significant association between the LI scores for non-paretic side movements and the FMA scale for individuals after stroke. This indicates that the FMA scale, which evaluates the movement function of the paretic side in comparison to the non-paretic side [62] did not show a significant relationship with the neuronal activations (i.e., LI scores) for non-paretic side movements.

In other application scenarios, neurostimulation such as tDCS has been shown to enhance interhemispheric functional connectivity (FC) in individuals with chronic stroke, with the FC strongly associated with upper extremity Fugl–Meyer assessment scores [8]. Our study provides valuable information regarding the cortical hotspot for the lower extremities that can be targeted to enhance their function using noninvasive neuromodulation techniques [7, 8, 63]. In addition, locomotor training such as the use of a treadmill has proven useful for improving gait function [64, 65]. However, determining the optimal onset time for these training sessions to ensure long-lasting, meaningful improvement may not be straightforward [64, 65]. Our findings can provide valuable information regarding when locomotion training needs to begin to maximize rehabilitation outcomes by monitoring the LI of each lower limb movement identified in this study. Future research can also monitor the cortical loci of each lower limb to determine whether there is any change in brain function using noninvasive brain stimulation techniques and/or locomotion training in the chronic stage of stroke [66].

It is also worth noting that lower limb motor recovery after stroke can be achieved via non-cortical neural mechanisms such as indirect motor pathways from the brain stem for gait and balance enhancement [67–70]. For example, robot-assisted gait training has been useful in improving balance-related motor function for patients with infratentorial stroke [67]. Specifically, the hyperexcitability of reticulospinal tracts and potentially vestibulospinal tracts may be an important mechanism underlying stroke-induced spasticity and disordered motor control, which can be further understood using functional neuroimaging such as fMRI [69]. In particular, our findings highlight the importance of the ipsilateral motor pathways for paretic lower limb movement and recovery [71] and offer potential suggestions for the strengthening/stretching of affected muscles in stroke rehabilitation to correct asymmetric postural patterns for gait control [72], thus providing broad insights into rehabilitation guidance.

### Limitations of the study

It is important to recognize the potential limitations and weaknesses of this study. First, power analysis indicated that 12 subjects was sufficient to achieve at least 80% power with alpha = 0.05. Nevertheless, considering the individual variability of fMRI patterns and current trends in fMRI studies, our sample size was relatively small. The reliability of our findings would be enhanced by using the dataset from a higher number of participants, possibly by taking specific age ranges and gender distributions between groups into consideration to minimize potential confounding factors that affect BOLD responses [73]. We also did not consider the specific assessment of lower limb motor function or strength in healthy subjects. However, we confirmed that their lower limb function/strength was substantially higher than the individuals after stroke based on WFQ-R (Table 1). Individual variability in neuronal activations may have occurred depending on the lower limb strength of healthy subjects based on previous reports on upper limb movement, with stronger neuronal activations observed for strong grasping compared with light grasping [74].

The ability of our approach to reduce potential head motion during fMRI acquisition was evaluated using 11 healthy subjects. Although we believe that a similar efficacy would be observed with a higher number of participants when using the equipment, the limited number of subjects used in the testing for our study represents a potential limitation. In addition, it would have been valuable to include individuals after stroke to assess the feasibility and effectiveness of head motion reduction for this group. However, we could not recruit individuals after stroke for this session. Furthermore, we could not collect additional fMRI data with and without the equipment from our recruited subjects with stroke due to the prolonged acquisition time and fatigue, which prevented them from performing additional task movements without the equipment.

Only some of the individuals after stroke completed all four movements for each side of the leg (six out of 15) and the remaining nine individuals after stroke completed only ankle dorsiflexion and toe flexion. Some of the individuals could not or struggled to perform ankle rotation and knee extensions in the MRI, thus we asked them to perform only the two lower limb movements. Therefore, there may have been significant variation in the lower limb functional status of our recruited individuals after stroke, which would have potentially affected the within-group findings. The decision to modify the EPI parameters for fMRI data acquisition to improve the signal-to-noise ratio of the BOLD contrast for individuals after stroke was made when we acquired the fMRI data from most of the healthy participants. This modification of the EPI parameters may compromise the comparability of the findings between and within groups.

Furthermore, our adopted lower extremity movements did not include movements initiated from the hip joint, which plays a significant role in gait function recovery after stroke [75]. This was partly because our MR-compatible equipment could not accommodate the hip joint movement and reduce corresponding head motion. Future studies should highlight the potential relevance of different hip joint movements in stroke rehabilitation by employing functional neuroimaging modality. We also only considered the PCL as an ROI to investigate potential hierarchical cortical loci associated with lower limb movement in the medial wall of the motor area. However, other potentially relevant brain regions with lower limb movements, such as the primary motor cortex, supplementary motor area, and cingulate gyrus, could be included for analysis [11, 76] along with whole brain analysis, including the cerebellum [61].

## Conclusion

We reported the cortical loci specific to four lower limb movements in the PCL using fMRI data recorded when participants engaged in toe flexion, ankle dorsiflexion, ankle rotation, and knee extension. We believe that the identification of the cortical loci for these four lower limb movements was possible due to our custom-made MR-compatible footplate (to isolate the individual lower limb movements of the ankle and toe and to reduce head motion) and leg cushion (to guide leg movement and further reduce head motion), and due to the multivariate analyses employed on the acquired fMRI dataset. The validity of the identified cortical loci was evaluated via cross-validation using an fMRI dataset acquired from independent healthy participants and LI scores from the identified cortical loci compared to the PCL. The efficacy of our fine-grained cortical loci for lower limb movements was also demonstrated for individuals after stroke, with the LI scores calculated from the identified cortical loci more strongly correlated with the FMA scale than the PCL was. We believe that our findings will benefit future studies that require the isolation of cortical loci for lower limb movements for neurorehabilitation purposes. For example, for individuals after stroke in an acute or sub-acute stage whose lower limb(s) are compromised due to stroke lesions, the efficiency of their rehabilitation could be improved with the help of the identified cortical loci by evaluating and monitoring the neuronal activations in the foci and their LI scores. Another possible scenario for individuals after stroke is to use neurorehabilitation to enhance the LI scores calculated based on the neuronal activations of the cortical loci on the paretic and non-paretic sides of the brain monitored with real-time fMRI-based data and/or neurofeedback [43, 77–80]. Our findings may also have potential practical implications for non-invasive brain stimulations such as rTMS and tDCS when looking to optimize rehabilitation programs to enhance lower limb functions [7, 8, 63].

## Data Availability

The data that support the findings of this study are available from the corresponding author upon reasonable request.

## References

[1] Horn U, Grothe M, Lotze M (2016). MRI biomarkers for hand-motor outcome prediction and therapy monitoring following stroke. Neural Plast.

[2] Strother L, Medendorp P, Coros A, Vilis T (2012). Double representation of the wrist and elbow in human motor cortex. Eur J Neurosci.

[3] Lee SH, Jin SH, An J (2019). The difference in cortical activation pattern for complex motor skills: a functional near- infrared spectroscopy study. Sci Rep.

[4] Stark A, Meiner Z, Lefkovitz R, Levin N (2012). Plasticity in cortical motor upper-limb representation following stroke and rehabilitation: two longitudinal multi-joint fMRI case-studies. Brain Topogr.

[5] Wanni Arachchige PR, Ryo U, Karunarathna S, Senoo A (2021). Evaluation of fMRI activation in hemiparetic stroke patients after rehabilitation with low-frequency repetitive transcranial magnetic stimulation and intensive occupational therapy. Int J Neurosci.

[6] Zhu M-H, Zeng M, Shi M-F, Gu X-D, Shen F, Zheng Y-P (2020). Visual feedback therapy for restoration of upper limb function of stroke patients. Int J Nurs Sci.

[7] Parikh V, Medley A, Chung Y-C, Goh H-T (2023). Optimal timing and neural loci: a scoping review on the effect of non-invasive brain stimulation on post-stroke gait and balance recovery. Top Stroke Rehabil.

[8] Unger RH, Lowe MJ, Beall EB, Bethoux F, Jones SE, Machado AG (2023). Stimulation of the premotor cortex enhances interhemispheric functional connectivity in association with upper limb motor recovery in moderate-to-severe chronic stroke. Brain Connect..

[9] Grooms DR, Diekfuss JA, Ellis JD, Yuan W, Dudley J, Foss KDB (2019). A novel approach to evaluate brain activation for lower extremity motor control. J Neuroimaging.

[10] Luft AR, Smith GV, Forrester L, Whitall J, Macko RF, Hauser T-K (2002). Comparing brain activation associated with isolated upper and lower limb movement across corresponding joints. Hum Brain Mapp.

[11] Kapreli E, Athanasopoulos S, Papathanasiou M, Van Hecke P, Strimpakos N, Gouliamos A (2006). Lateralization of brain activity during lower limb joints movement. An fMRI study. Neuroimage.

[12] Kapreli E, Athanasopoulos S, Papathanasiou M, Van Hecke P, Kelekis D, Peeters R (2007). Lower limb sensorimotor network: issues of somatotopy and overlap. Cortex.

[13] Orr ELR, Lacourse MG, Cohen MJ, Cramer SC (2008). Cortical activation during executed, imagined, and observed foot movements. NeuroReport.

[14] Villiger M, Estévez N, Hepp-Reymond MC, Kiper D, Kollias SS, Eng K (2013). Enhanced activation of motor execution networks using action observation combined with imagination of lower limb movements. PLoS ONE.

[15] Kline A, Pittman D, Ronsky J, Goodyear B (2020). Differentiating the brain’s involvement in executed and imagined stepping using fMRI. Behav Brain Res.

[16] Catani M (2017). A little man of some importance. Brain.

[17] Ciccarelli O, Toosy AT, Marsden JF, Wheeler-Kingshott CM, Sahyoun C, Matthews PM (2005). Identifying brain regions for integrative sensorimotor processing with ankle movements. Exp Brain Res.

[18] Mehta JP, Verber MD, Wieser JA, Schmit BD, Schindler-Ivens SM (2009). A novel technique for examining human brain activity associated with pedaling using fMRI. J Neurosci Methods.

[19] Sahyoun C, Floyer-Lea A, Johansen-Berg H, Matthews PM (2004). Towards an understanding of gait control: brain activation during the anticipation, preparation and execution of foot movements. Neuroimage.

[20] Jaeger L, Marchal-Crespo L, Wolf P, Riener R, Michels L, Kollias S (2014). Brain activation associated with active and passive lower limb stepping. Front Human Neurosci.

[21] Bürki CN, Bridenbaugh SA, Reinhardt J, Stippich C, Kressig RW, Blatow M (2017). Imaging gait analysis: an fMRI dual task study. Brain Behav.

[22] Newton JM, Dong Y, Hidler J, Plummer-D’Amato P, Marehbian J, Albistegui-DuBois RM (2008). Reliable assessment of lower limb motor representations with fMRI: use of a novel MR compatible device for real-time monitoring of ankle, knee and hip torques. Neuroimage.

[23] Patra A, Kaur H, Chaudhary P, Asghar A, Singal A (2021). Morphology and morphometry of human paracentral lobule: an anatomical study with its application in neurosurgery. Asian J Neurosurg.

[24] Mur M, Bandettini PA, Kriegeskorte N (2009). Revealing representational content with pattern-information fMRI—an introductory guide. Soc Cogn Affect Neurosci.

[25] Kriegeskorte N, Mur M, Bandettini P (2008). Representational similarity analysis - connecting the branches of systems neuroscience. Front Syst Neurosci.

[26] Szucs D, Ioannidis JPA (2020). Sample size evolution in neuroimaging research: an evaluation of highly-cited studies (1990–2012) and of latest practices (2017–2018) in high-impact journals. Neuroimage.

[27] Faul F, Erdfelder E, Lang A-G, Buchner A (2007). G*Power 3: a flexible statistical power analysis program for the social, behavioral, and biomedical sciences. Behav Res Methods.

[28] Faul F, Erdfelder E, Buchner A, Lang AG (2009). Statistical power analyses using G*Power 3.1: tests for correlation and regression analyses. Behav Res Methods.

[29] Park J-H, Kwon YC (1990). Modification of the mini-mental state examination for use in the elderly in a non-western society. Part 1. Development of korean version of mini-mental state examination. Int J Geriatr Psychiatry.

[30] Jackson-Koku G (2016). Beck depression inventory. Occup Med.

[31] Beck Anxiety Inventory—PsycNET. https://psycnet.apa.org/doiLanding?doi=10.1037%2Ft02025-000. Accessed 30 Aug 2022.

[32] Big Five Inventory—PsycNET. https://psycnet.apa.org/doiLanding?doi=10.1037%2Ft07550-000. Accessed 30 Aug 2022.

[33] Löwe B, Unützer J, Callahan CM, Perkins AJ, Kroenke K (2004). Monitoring depression treatment outcomes with the patient health questionnaire-9. Med Care.

[34] Oldfield RC (1971). The assessment and analysis of handedness: the Edinburgh inventory. Neuropsychologia.

[35] Elias LJ, Bryden MP, Bulman-Fleming MB (1998). Footedness is a better predictor than is handedness of emotional lateralization. Neuropsychologia.

[36] Fugl-Meyer AR, Jääskö L, Leyman I, Olsson S, Steglind S (1975). The post-stroke hemiplegic patient. 1. a method for evaluation of physical performance. Scand J Rehabil Med.

[37] Platz T, Pinkowski C, Van Wijck F, Kim I-H, Di Bella P, Johnson G (2005). Reliability and validity of arm function assessment with standardized guidelines for the Fugl-Meyer test, action research arm test and box and block test: a multicentre study. Clin Rehabil.

[38] Sanford J, Moreland J, Swanson LR, Stratford PW, Gowland C (1993). Reliability of the Fugl-Meyer assessment for testing motor performance in patients following stroke. Phys Ther.

[39] Duncan PW, Propst M, Nelson SG (1983). Reliability of the Fugl-Meyer assessment of sensorimotor recovery following cerebrovascular accident. Phys Ther.

[40] Caballero-Gaudes C, Reynolds RC (2017). Methods for cleaning the BOLD fMRI signal. Neuroimage.

[41] Ashby FG (2019). Statistical analysis of fMRI Data.

[42] Kim H-C, Bandettini PA, Lee J-H (2019). Deep neural network predicts emotional responses of the human brain from functional magnetic resonance imaging. Neuroimage.

[43] Kim H-C, Tegethoff M, Meinlschmidt G, Stalujanis E, Belardi A, Jo S (2019). Mediation analysis of triple networks revealed functional feature of mindfulness from real-time fMRI neurofeedback. Neuroimage.

[44] Chen G, Taylor PA, Shin Y-W, Reynolds RC, Cox RW (2017). Untangling the relatedness among correlations, part II: Inter-subject correlation group analysis through linear mixed-effects modeling. Neuroimage.

[45] Tzourio-Mazoyer N, Landeau B, Papathanassiou D, Crivello F, Etard O, Delcroix N (2002). Automated anatomical labeling of activations in SPM using a macroscopic anatomical parcellation of the MNI MRI single-subject brain. Neuroimage.

[46] Nichols TE, Holmes AP (2002). Nonparametric permutation tests for functional neuroimaging: a primer with examples. Hum Brain Mapp.

[47] Matsuo K, Chen S-HA, Tseng W-YI (2012). AveLI: A robust lateralization index in functional magnetic resonance imaging using unbiased threshold-free computation. J Neurosci Methods.

[48] Akselrod M, Martuzzi R, Serino A, van der Zwaag W, Gassert R, Blanke O (2017). Anatomical and functional properties of the foot and leg representation in areas 3b, 1 and 2 of primary somatosensory cortex in humans: A 7T fMRI study. Neuroimage.

[49] Kriegeskorte N, Mur M, Ruff DA, Kiani R, Bodurka J, Esteky H (2008). Matching categorical object representations in inferior temporal cortex of man and monkey. Neuron.

[50] Norman KA, Polyn SM, Detre GJ, Haxby JV (2006). Beyond mind-reading: multi-voxel pattern analysis of fMRI data. Trends Cogn Sci.

[51] Kim D-Y, Jung EK, Zhang J, Lee S-Y, Lee J-H (2020). Functional magnetic resonance imaging multivoxel pattern analysis reveals neuronal substrates for collaboration and competition with myopic and predictive strategic reasoning. Hum Brain Mapp.

[52] Saadon-Grosman N, Loewenstein Y, Arzy S (2020). The “creatures” of the human cortical somatosensory system multiple somatosensory homunculi. Brain Commun..

[53] Muret D, Root V, Kieliba P, Clode D, Makin TR (2022). Beyond body maps: Information content of specific body parts is distributed across the somatosensory homunculus. Cell Rep.

[54] Kim H-C, Jin S, Jo S, Lee J-H (2020). A naturalistic viewing paradigm using 360° panoramic video clips and real-time field-of-view changes with eye-gaze tracking. Neuroimage.

[55] Levy CE, Nichols DS, Schmalbrock PM, Keller P, Chakeres DW (2001). Functional MRI evidence of cortical reorganization in upper-limb stroke hemiplegia treated with constraint-induced movement therapy. Am J Phys Med Rehab.

[56] Binder E, Leimbach M, Pool E-M, Volz LJ, Eickhoff SB, Fink GR (2021). Cortical reorganization after motor stroke: A pilot study on differences between the upper and lower limbs. Hum Brain Mapp.

[57] Luft AR, Forrester L, Macko RF, McCombe-Waller S, Whitall J, Villagra F (2005). Brain activation of lower extremity movement in chronically impaired stroke survivors. Neuroimage.

[58] Caglayan AB, Beker MC, Caglayan B, Yalcin E, Caglayan A, Yulug B (2019). Acute and post-acute neuromodulation induces stroke recovery by promoting survival signaling, neurogenesis, and pyramidal tract plasticity. Front Cell Neurosci.

[59] Grefkes C, Fink GR (2020). Recovery from stroke: current concepts and future perspectives. Neurol Res Pract.

[60] Enzinger C, Johansen-Berg H, Dawes H, Bogdanovic M, Collett J, Guy C (2008). Functional MRI correlates of lower limb function in stroke victims with gait impairment. Stroke.

[61] Ward NS, Brown MM, Thompson AJ, Frackowiak RS (2003). Neural correlates of motor recovery after stroke: a longitudinal fMRI study. Brain.

[62] Gladstone DJ, Danells CJ, Black SE (2002). The Fugl-Meyer assessment of motor recovery after stroke: a critical review of its measurement properties. Neurorehabili Neural Repair.

[63] Sivaramakrishnan A, Tahara-Eckl L, Madhavan S (2016). Spatial localization and distribution of the TMS-related ‘hotspot’ of the tibialis anterior muscle representation in the healthy and post-stroke motor cortex. Neurosci Lett.

[64] Spiess MR, Steenbrink F, Esquenazi A (2018). Getting the best out of advanced rehabilitation technology for the lower limbs: minding motor learning principles. PM&R.

[65] Forrester LW (2008). Exercise-mediated locomotor recovery and lower-limb neuroplasticity after stroke. JRRD.

[66] Liepert J, Bauder H, Miltner WHR, Taub E, Weiller C (2000). Treatment-induced cortical reorganization after stroke in humans. Stroke.

[67] Kim HY, Shin J-H, Yang SP, Shin MA, Lee SH (2019). Robot-assisted gait training for balance and lower extremity function in patients with infratentorial stroke: a single-blinded randomized controlled trial. J NeuroEngineering Rehabil.

[68] Suh JH, Han SJ, Jeon SY, Kim HJ, Lee JE, Yoon TS (2014). Effect of rhythmic auditory stimulation on gait and balance in hemiplegic stroke patients. NeuroRehabilitation.

[69] Li S, Francisco GE (2015). New insights into the pathophysiology of post-stroke spasticity. Front Hum Neurosci.

[70] Li S, Chen Y-T, Francisco GE, Zhou P, Rymer WZ (2019). A unifying pathophysiological account for post-stroke spasticity and disordered motor control. Front Neurol.

[71] Cleland BT, Madhavan S (2021). Ipsilateral motor pathways to the lower limb after stroke: insights and opportunities. J Neurosci Res.

[72] Beyaert C, Vasa R, Frykberg GE (2015). Gait post-stroke: pathophysiology and rehabilitation strategies. Clin Neurophysiol.

[73] Tsvetanov KA, Henson RNA, Jones PS, Mutsaerts H, Fuhrmann D, Tyler LK (2021). The effects of age on resting-state BOLD signal variability is explained by cardiovascular and cerebrovascular factors. Psychophysiology.

[74] Kwon HG, Kim JS, Lee MY (2019). Brain activation induced by different strengths of hand grasp: a functional magnetic resonance imaging study. Neural Regen Res.

[75] Lee HS, Ryu H, Lee SU, Cho JS, You S, Park JH, Jang SH (2021). Analysis of gait characteristics using hip-knee cyclograms in patients with hemiplegic stroke. Sensors.

[76] Frías I, Starrs F, Gisiger T, Minuk J, Thiel A, Paquette C (2018). Interhemispheric connectivity of primary sensory cortex is associated with motor impairment after stroke. Sci Rep.

[77] Wang T, Mantini D, Gillebert CR (2018). The potential of real-time fMRI neurofeedback for stroke rehabilitation: a systematic review. Cortex.

[78] Liew S-L, Rana M, Cornelsen S, de Fortunato Barros Filho M, Filho M, Birbaumer N, Sitaram R (2016). Improving motor corticothalamic communication after stroke using real-time fMRI connectivity-based neurofeedback. Neurorehabil Neural Repair.

[79] Kim D-Y, Yoo S-S, Tegethoff M, Meinlschmidt G, Lee J-H (2015). The inclusion of functional connectivity information into fMRI-based neurofeedback improves its efficacy in the reduction of cigarette cravings. J Cogn Neurosci.

[80] Lee J-H, O’Leary HM, Park H, Jolesz FA, Yoo S-S (2008). Atlas-based multichannel monitoring of functional MRI signals in real-time: automated approach. Human Brain Mapp.

